# Mechanism of Forelimb Motor Function Restoration after Cervical Spinal Cord Hemisection in Rats: A Comparison of Juveniles and Adults

**DOI:** 10.1155/2016/1035473

**Published:** 2016-01-28

**Authors:** Atsushi Hasegawa, Masahito Takahashi, Kazuhiko Satomi, Hideaki Ohne, Takumi Takeuchi, Shunsuke Sato, Shoichi Ichimura

**Affiliations:** ^1^Department of Orthopaedic Surgery, Kyorin University, 6-20-2 Shinkawa, Mitaka-shi, Tokyo 181-0004, Japan; ^2^Department of Orthopaedic Surgery, Kugayama Hospital, 2-14-20 Kitakarasuyama, Setagaya-ku, Tokyo 157-0061, Japan

## Abstract

The aim of this study was to investigate forelimb motor function after cervical spinal cord injury in juvenile and adult rats. Both rats received a left segmental hemisection of the spinal cord after C3-C4 laminectomy. Behavioral evaluation of motor function was monitored and assessed using the New Rating Scale (NRS) and Forelimb Locomotor Scale (FLS) and by measuring the range of motion (ROM) of both the elbow and wrist. Complete left forelimb motor paralysis was observed in both rats. The NRS showed motor function recovery restored to 50.2 ± 24.7% in juvenile rats and 34.0 ± 19.8% in adult rats. FLS was 60.4 ± 26.8% in juvenile rats and 46.5 ± 26.9% in adult rats. ROM of the elbow and wrist were 88.9 ± 20.6% and 44.4 ± 24.1% in juvenile rats and 70.0 ± 29.2% and 40.0 ± 21.1% in adult rats. Thus, the NRS and ROM of the elbow showed a significant difference between age groups. These results indicate that left hemisection of the cervical spinal cord was not related to right-sided motor functions. Moreover, while motor paralysis of the left forelimb gradually recovered in both groups, the improvement was greater in juvenile rats.

## 1. Introduction

After the report by Cajal et al. in 1928, which purported that, once injured, the mammalian central nervous system (CNS) cannot regenerate, a number of studies were published that supported this contention [1–3]. The observations in these studies suggested that axonotmesis occurs in the damaged brain and spinal cord (primary damage) and that even cells of the surrounding area undergo necrosis and apoptosis forming a cavity (secondary injury). After the inflammation due to injury subsides, it is thought that the spinal cord does not regenerate because the glial scar that forms around the cavity inhibits the elongation and regeneration of axons [4]. Therefore, in order to restore the function of nerves after spinal cord injury, it is presently thought that it is necessary to suppress secondary damage (axonal regeneration after glial scar formation), develop regenerative nervous medicine, and restore the remaining CNS.

Recently, in reference to the injured spinal cord, there has been an increase in the number of studies that aim to suppress secondary damage through cell transplantation such as neural stem cells, embryonic stem cells (ES cells), and induced pluripotent stem cells (iPS cells), thus promoting axonal regeneration and reconstruction of neuronal circuits [5]. On the other hand, other studies have focused on the restoration of the remaining nerves, such as reorganization of the corticospinal tract on the uninjured side after damage to the corticospinal tract of juvenile rats [6]. Further, various experimental studies have shown restoration of hindlimb motor function in rats, even after complete damage to the thoracic spinal cord [7]. Much, however, remains to be clarified concerning the reported restoration of forelimb motor function after cervical spinal cord or brain injury. Recently, there has been an increase in the number of studies evaluating forelimb motor function after damage to the cervical spinal cord of rats, based on impact that is similar to cervical spinal cord injury in humans [8]. However, with this approach, it is possible that the spinal cord on the uninjured side might be damaged. Therefore, it is difficult to accurately judge which route to activate and whether motor functions have in fact recovered. One study demonstrated that a substantial change in the route of the corticospinal tract occurred in juvenile rats after brain injury and, furthermore, that the animals paralyzed because of the injury showed recovery of forelimb motor function after the change [6]. It thus appears that although the CNS does not regenerate once damaged, formation of an alternate neuronal pathway can compensate for impaired motor function. Consequently, in order to properly evaluate the compensatory function of the nervous system on the uninjured side, it is necessary to perform spinal cord hemisection; however, such research has not yet been conducted. In the current study, we performed a complete hemisection of the corticospinal tract on the injured side following damage by cutting exactly half of the cervical spinal cord at the upper cervical spinal cord level. This allowed us to produce a hemisection rat model that disrupted the forelimb and hindlimb motor functions. Using this approach, we were able to verify whether forelimb motor function recovered on the injured side not only after damage to the cerebral cortex spinal tract, but also after injury to the cervical spinal cord via the route change of the corticospinal tract on the uninjured side. Evaluations were first conducted on juvenile rats in which the compensatory mechanism of nerves occurs easily. If functional restoration was observed, adult rats were prepared using a similar condition of injury, and we then verified whether there was a difference in functional restoration between the two groups.

In conclusion, we would like to note that the purpose of this study was not to contribute to regenerative medicine, but to compensatory medicine. Furthermore, a highly detailed understanding of how undamaged neurons are altered following injury is necessary for the implementation of regenerative and/or compensatory medicine.

## 2. Materials and Methods

Juvenile and adult rat models of spinal cord injury were established by left segmental hemisection of the cervical spinal cord after a C3-C4 laminectomy. Forelimb motor functions in these animal models were then examined over a period of 6 weeks by three different methods: electrophysiological, histopathological, and assessment and statistical comparison of forelimb motor function restoration between juvenile and adult rats.

### 2.1. Animals

A total of 33 male Wistar rats were used. Rats used included 21 three-week-old juveniles weighing from 60 to 90 g and 12 twelve-week-old adults weighing from 350 to 400 g.

### 2.2. Surgery-Spinal Cord Hemisection

**Figure 1:**
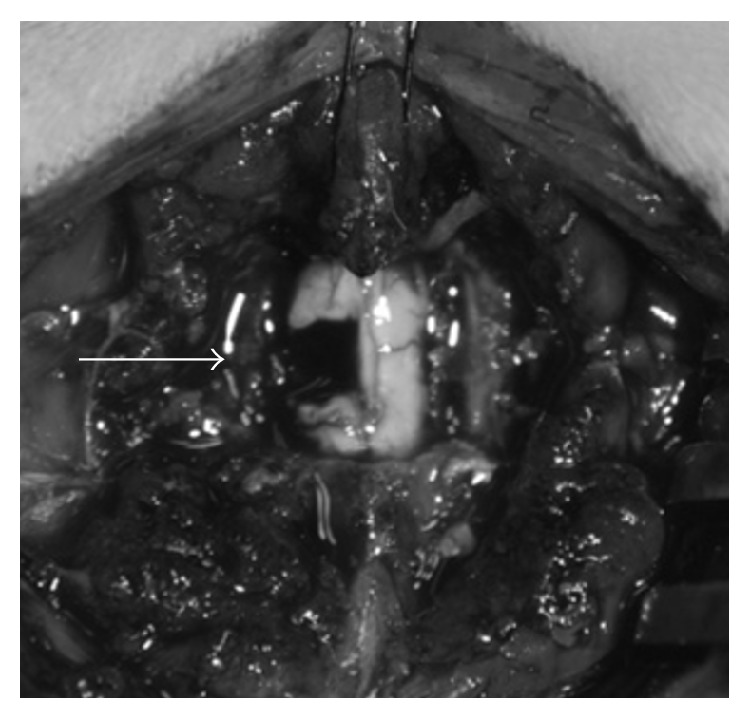
Photograph of the C2−C5 laminae. White Arrow: C3/C4 spinal cord hemisection showing a 2 mm gap.

Animals were first anesthetized with intraperitoneal xylazine (10 mg/kg; Bayer Health Care, Monheim, Germany) and ketamine (90 mg/kg; Daiichi Sankyo, Tokyo, Japan). For intraoperative monitoring of vital signs, rectal temperature, arterial oxygen saturation, heart rate, and respiratory rate were continuously measured. With the rat restrained on a stereotaxic instrument, the C2–C5 laminae were exposed via a posterior approach, allowing a C3-C4 laminectomy to be carried out, followed by exposure of the dura mater. After both the dura mater and arachnoid membrane were incised to expose the posterior aspect of the spinal cord, a left hemisection of the cervical spinal cord was performed after confirming fibers of the C3 and C4 root. The spinal cord hemisection was carried out segmentally over a width of 2 mm in order to prevent cutting remainder in the hemisectioned region and to avoid neuronal readhesions and elongation of the injured axons in that region (Figure 1). The animals were administered subcutaneous (s.c.) injections of buprenorphine (0.02 mg/kg; Otsuka, Tokyo, Japan) at 12-hour intervals for 3 days as postoperative analgesia, in addition to intramuscular injections of penicillin G (22 000 units/kg; Tamura, Tokyo, Japan) once every 24 hours for 3 days as antimicrobial prophylaxis.

### 2.3. Evaluation of the Spinal Cord Hemisection

**Figure 2:**
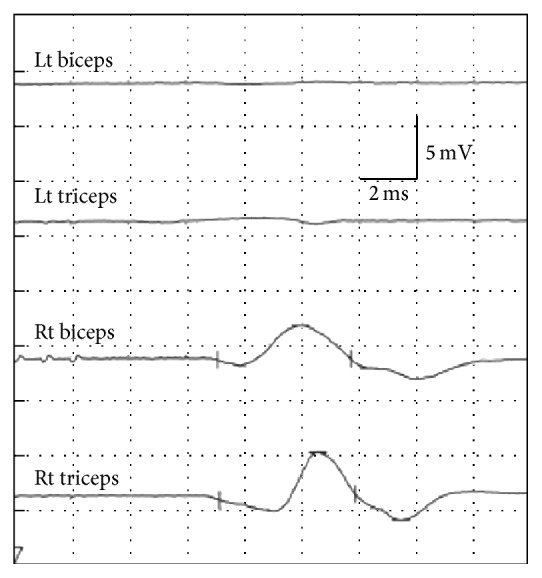
Changes in CMAP of the bilateral biceps and triceps after spinal cord hemisection. Amplitudes of the left biceps and triceps were disappointed.

*Electrophysiological Examinations*. In response to bilateral transcranial stimulation via needle electrodes inserted 1.5 cm s.c., compound muscle action potentials (CMAPs) evoked in the left and right bicep brachii and triceps brachii muscles were measured. The conditions of the stimulation were as follows: three continuous stimulations (2 ms interval), 50 mA intensity, and a 1 Hz rate. The waveforms of the responses were recorded on a Neuropack MEB-2200 (Nihon Kohden Corporation, Tokyo, Japan). Disappearance of the response amplitudes on the injured side alone upon cervical spinal cord hemisection was confirmed (Figure 2).

### 2.4. Pathology

After completion of the behavioral assessment, rats were sacrificed by phosphate-buffered saline (PBS: 0.9% NaCl + 0.1 M phosphate buffer) infusion under general anesthesia induced by intraperitoneal pentobarbital sodium (50 mg/kg) and fixed by perfusion with 4% paraformaldehyde in PBS. A spinal cord specimen about 10 mm in length primarily including the injured region was cut from each rat, frozen, and embedded using a 30% sucrose solution. This was followed by 20 *μ*m thin sectioning with a cryostat microtome and examination by light microscopy after toluidine blue staining. It was confirmed through this procedure that the cervical spinal cord hemisection had been accomplished appropriately in all the rats in which the behavioral assessment was completed.

### 2.5. Behavioral Tests

As a preparatory step for the evaluation of forelimb motor function, we set up a testing environment that would allow straight locomotion of the rat. This was achieved with the use of an acrylic board box providing a space measuring 180 cm (*L*) × 20 cm (*W*) × 40 cm (*H*), with a slip-proof sheet spread over the floor. The behavior of each animal in the acrylic box was videotaped for approximately 4 minutes using a video camera (Victor JVC, GZ-X900), and if the animal stayed unmoving at any single point for 20 seconds or longer, it was returned to the starting point and the videotaping was continued. The evaluation was performed prior to the cord hemisection and on days 1, 2, 5, 7, 14, 21, 28, 35, and 42 after the cord hemisection. Scoring in these qualitative behavioral assessments was performed from prior to the cord hemisection until day 42 after the cord hemisection and was carried out by four observers. The mean of the second and third of the four scorings was adopted as the score for inclusion in the analysis.

#### 2.5.1. New Rating Scale [9]

In this behavioral evaluation, the motor functions of the fore- and hindlimbs of each rat were rated on a 0–20-point scale based on the (1) range of joint motion, (2) body weight support, (3) condition of the digits, (4) walking/gait, (5) fore- and hindlimb coordinated movements, and (6) tail position; and only the scores for the forelimbs were adopted for the analysis (Figure 9).

#### 2.5.2. Forelimb Locomotor Scale (FLS) [10]

Forelimb motor function was rated on a 0–17-point scale formulated on the basis of the Beattie and Bresnahan (BBB) locomotor rating scale [11] and was based on the (1) range of joint motion, (2) body weight support, and (3) balance between the trunk and the forelimb (Table 1).

#### 2.5.3. Range of Motion (ROM)

**Figure 3:**
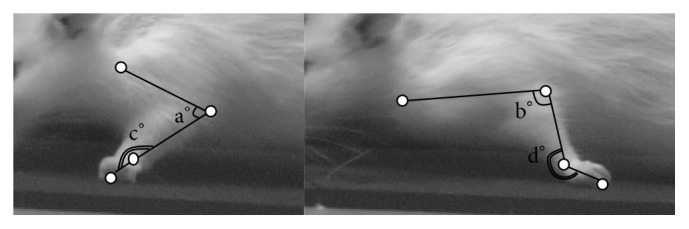
Range of motion (elbow: a–b°; wrist: c–d°).

For evaluating the recovery of muscle strength and joint motions of the forelimb in the rat models of cervical spinal cord hemisection, the difference in the ROM of the cubital and carpal joints of the forelimb between maximum extension and maximum flexion during the videotaped walking was calculated. The forelimb joint motion on the affected side was rated on a 0–3-point scale as follows: no joint motion (score = 0); less than 45° (score = 1); more than 45° but less than 90° (score = 2); more than 90° (score = 3) (Figure 3).

### 2.6. Statistical Analysis

The behavioral test data on the left and right forelimbs were statistically evaluated by an analysis of variance, and the recovery of the locomotor function between juvenile and adult rats was compared according to Mann-Whitney *U* test. The level of significance was set at *P* < 5%.

## 3. Results

### 3.1. Surgery-Spinal Cord Hemisection

**Figure 4:**
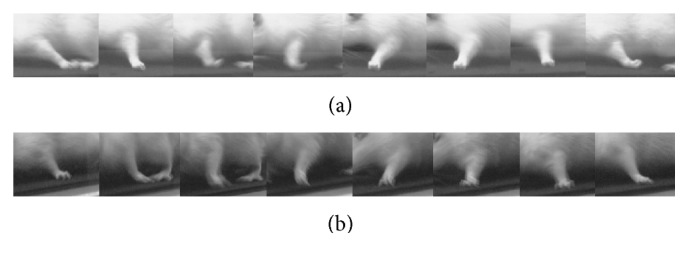
Sequential photographs of left forelimb movement on day 42 after hemisection. (a) Juvenile rat. (b) Adult rat.

Of the 33 rats subjected to spinal cord hemisection, 25 (including 15 juvenile rats and 10 adult rats) could be secured. Four of the eight rats excluded from the study died during the spinal cord hemisection, and, of the remaining four, two had residual posterior funiculus after the spinal cord hemisection, one developed quadriplegia due to the spinal cord hemisection extending to the opposite half of the cord, and one died of unknown causes on day 10 after the operation. After awakening from anesthesia, none of the 25 rats included in the study showed motor paralysis of the right forelimb, whereas complete flaccid motor paralysis was evident in the left forelimb; the motor paresis persisted until the final observation (Figures 4(a) and 4(b)).

**Figure 5:**
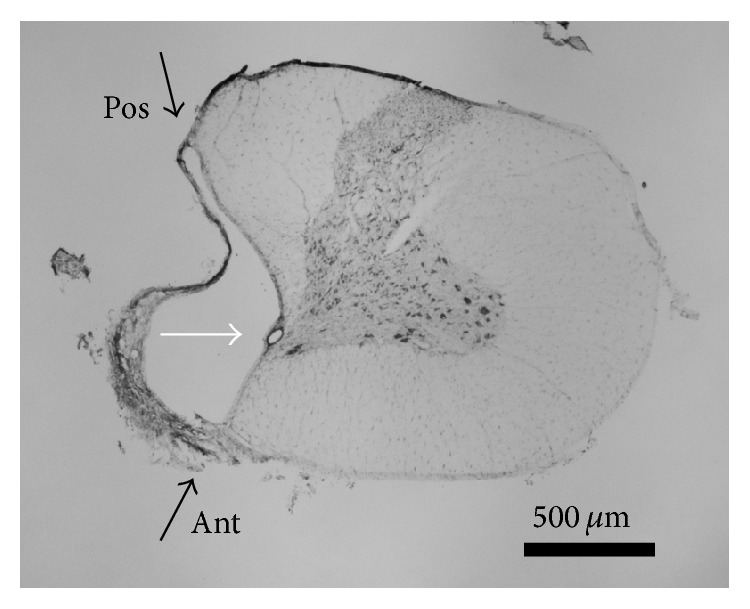
Microphotograph of the spinal cord at lesion level (C3/C4). Scale bar = 500 *μ*m. Black arrow: spinal cord midline. White arrow: central canal.

Histological examination revealed an intact right half of the cervical spinal cord without evidence of any scar or cavity formation. In addition to the hemisection being leftward, it occurred precisely from the median line (Figure 5).

### 3.2. Behavioral Tests

#### 3.2.1. New Rating Scale

**Figure 6:**
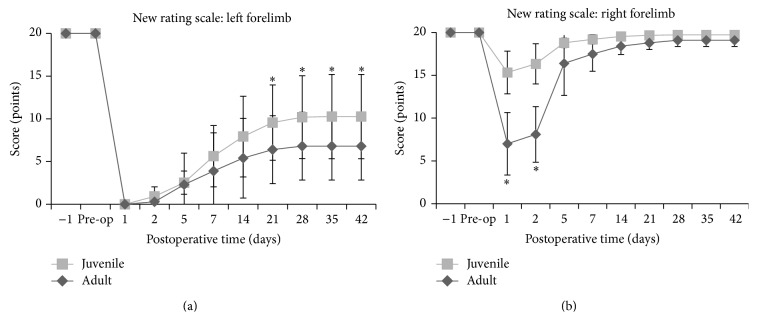
New Rating Scale scores in juvenile and adult rats. The New Rating Scale of the left forelimb shows that juvenile rats recovered significantly more than adult rats from day 21 (^*∗*^ *P* < 0.05). In contrast, the New Rating Scale of the right forelimb illustrates that no significant difference was seen between juvenile and adult rats from day 5.

Regarding left forelimb (affected side) motor function recovery, the rating scores in juvenile and adult rats were 0.7 ± 1.0 (0–3) and 0.3 ± 0.9 (0–3) on day 2 after the cord hemisection, 5.6 ± 3.6 (1–12) and 3.1 ± 3.7 (0–13) on day 7, and 10.2 ± 4.8 (3–17) and 6.1 ± 3.8 (3–13) on day 28 after the cord hemisection, respectively. Hence, while some residual motor paralysis of the left forelimb was observed, motor function was restored to 50.2 ± 24.7% in the juvenile rats and to 34.0% ± 19.8% in the adult rats. Thus, the motor function recovery of the left forelimb was significantly greater in the juvenile rats as compared to that in the adult rats (*P* = 0.03) (Figure 6). The rating scores, on the other hand, for motor function of the right forelimb (unaffected side) on day 42 after cord hemisection in juvenile and adult rats were 19.5 ± 0.5 (19-20) and 19.1 ± 0.7 (18–20), respectively. These findings indicate that there was significant difference between juvenile and adult rats in the motor function of the right forelimb, as well as the absence of any motor paralysis.

#### 3.2.2. FLS

**Figure 7:**
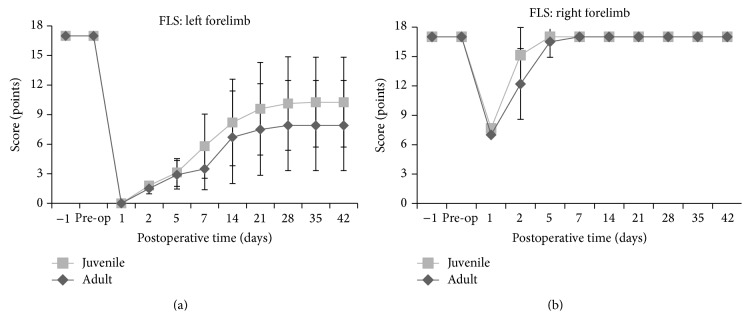
Forelimb Locomotor Scale (FLS) scores in juvenile and adult rats. The FLS of the left and right forelimb shows that no significant difference was noticeable between juvenile and adult rats.

The scores for the left forelimb (affected side) in juvenile and adult rats were 1.8 ± 0.4 (0–3) and 1.5 ± 0.5 (0–2) on day 2 after the cord hemisection, showed improvement to 5.8 ± 3.3 (2–12) and 3.5 ± 2.1 (2–7) on day 7, and were 10.1 ± 4.7 (2–17) and 7.9 ± 4.6 (2–15) on day 28 after the cord hemisection, respectively. The FLS score for the right forelimb (unaffected side), on the other hand, was restored to 17 in all juvenile and adult rats by day 7 after cord hemisection and remained at that level thereafter. Specifically, juvenile rats showed functional recovery to 60.4 ± 26.8% and adult rats showed functional recovery to 46.5% ± 26.9%, as assessed using the FLS rating criteria. There was no significant difference between juvenile and adult rats on day 42 after cord hemisection, that is, on the last day of behavioral evaluation (*P* = 0.06), although the recovery seemed slightly greater in juvenile rats (Figure 7).

#### 3.2.3. ROM

**Figure 8:**
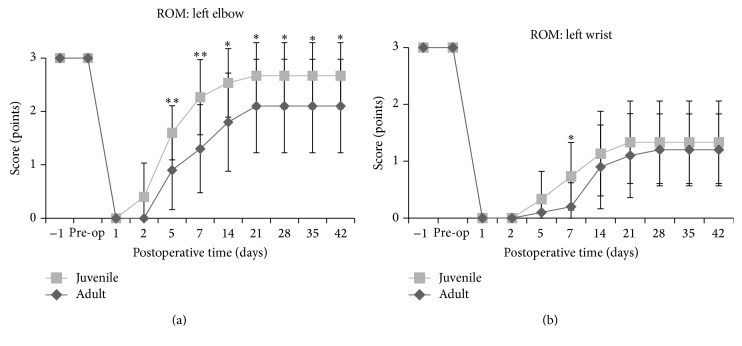
Range of motion (ROM) scores in juvenile and adult rats. The ROM of the left elbow shows that juvenile rats recovered significantly more than adult rats. The ROM score of the left wrist shows no significant difference between juvenile and adult rats (^*∗*^ *P* < 0.05; ^*∗∗*^ *P* < 0.01).

**Figure 9:**
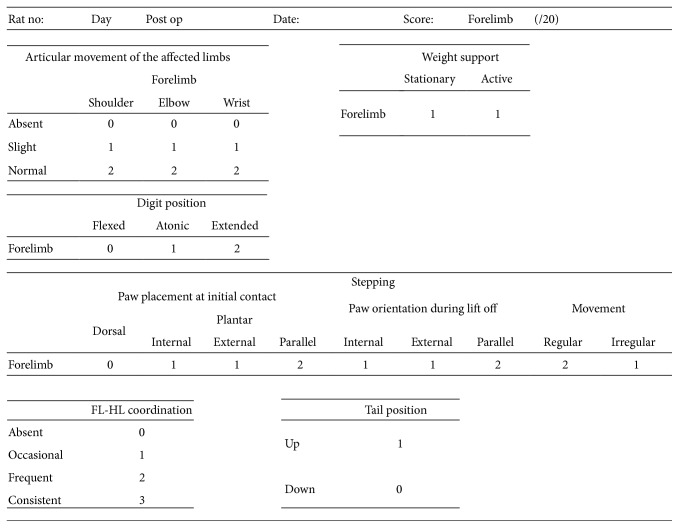
New Rating Scale: methods of scoring sheet (FL-HL, forelimb-hindlimb). (1) Range of motion of the shoulder, elbow, and wrist; (2) weight support of the forelimb; (3) state of the fingers and toes; forelimb walking motion; (4) stepping of the forelimb; (5) coordination of the forelimb-hindlimb; and (6) tail position.

In juvenile and adult rats, the ROM rating scores for the cubital joint were 0.4 ± 0.6 (0–2) and 0.0 ± 0.0 (0) on day 2 after the cord hemisection, 2.3 ± 0.7 (1–3) and 1.3 ± 0.8 (1–3) on day 7, and 2.7 ± 0.6 (1–3) and 2.1 ± 0.8 (1–3) on day 42 after the cord hemisection, respectively. The scores in juvenile and adult rats for the carpal joint were 0.0 ± 0.0 (0) and 0.0 ± 0.0 (0) on day 2 after cord hemisection, 0.7 ± 0.6 (0–2) and 0.2 ± 0.4 (0-1) on day 7, and 1.3 ± 0.7 (0–3) and 1.2 ± 0.4 (0–2) on day 42 after the cord hemisection, respectively. Thus, the cubital joint showed a ROM recovery to 88.9 ± 20.6% and the carpal joint a ROM recovery to 44.4 ± 24.1% in juvenile rats. The corresponding percentages for the function recovery of the cubital and carpal joints in adult rats were 70.0 ± 29.2% and 40.0 ± 21.1%, respectively (Figure 8). A comparison of the recoveries between juvenile and adult rats on the last day of behavioral evaluation revealed a significantly different degree of functional recovery for the cubital joint (*P* = 0.03), but not for the carpal joint (*P* = 0.32). Furthermore, in both juvenile and adult rats, there was a marked difference in the degree of functional recovery between the cubital and carpal joints.

## 4. Discussion

It is universally recognized that following injury to the mammalian CNS, the nerves of the CNS do not regenerate or become restored, and the paralysis that results from the injury does not improve [1]. However, various animal experiments have demonstrated that the motor function of an organ innervated by an injured central nerve is often restored, although the prevailing belief is still that the injured central nerve itself shows no spontaneous regeneration or restoration [7, 12]. In this research, a rat model of a cervical spinal cord hemisection was prepared for the evaluation of the remaining nervous system after cervical spinal cord injury.

In humans, a lesion in either lateral half of the spinal cord leads to the development of a paralysis called Brown-Séquard's syndrome. The syndrome is characterized by motor paralysis at and below the level of the spinal cord injury due to interruption of the corticospinal tract that courses through the lateral funiculus of the spinal cord, while sensory paralysis occurs on the opposite side when either half of the spinal cord is disrupted. A similar paralysis may also arise in the rat, although the pathway of the rat corticospinal tract differs from that in humans [13]. It has been verified that, in rats, the corticospinal tract courses closer to the central cord region and through the deep posterior funiculus; this difference aside, we confirmed that the rats in this study presented with a paralysis essentially similar to that observed in humans [6]. This was evident since, following complete injury of the posterior funiculus via a precise, left hemisection of the cervical spinal cord, the animals developed motor paralysis of the fore- and hindlimbs on the affected side (left), while the motor function on the opposite side (right) remained intact. In other words, since there was no injury to the right posterior funiculus, there was no motor paralysis present in the right fore- and hindlimbs. In this research, the cervical spinal cords of model rats were precisely cut in half through histopathological evaluation. Conversely, it was confirmed that the right spinal cord on the uninjured side was not damaged. In reference to forelimb motor function that could be attributed to the remainder of the cervical spinal cord [6], only muscles of the left upper extremity (ipsilateral to the side of injury) disappeared in the CMAP immediately following the hemisection. In addition, the motion of the forelimb on the injured side 1 day after hemisection was not seen because it displayed paralysis.

The present experiment demonstrated that motor function in the completely paralyzed forelimb was partly restored in all juvenile and adult rats, as assessed by behavioral tests. In the context of the score on the last day of observation, the degree of recovery in juvenile and adult rats was 50.2 ± 24.7% and 34.0 ± 19.8%, respectively, as assessed by the New Rating Scale, and 60.4 ± 26.8% and 46.5 ± 26.9%, respectively, as assessed by the FLS. Therefore, it was confirmed that both groups, to some extent, experienced a recovery of motion paralysis and that juvenile rats exhibited greater recovery. It can thus be concluded that some neural compensatory mechanism was involved in the restoration of forelimb motor function in the present experimental study. We speculate that the compensatory mechanism could be based on the following points. First, a review of the literature on motor function restoration through a neural compensatory mechanism revealed sporadic reports describing the presence of a nerve circuit different from the ordinary corticospinal tract. According to one report, hindlimb motor function was restored as a result of an increase in spinal cord crossover fibers in animals following thoracic spinal cord hemisection [14]. Another study noted projections to the contralateral motoneuronal nucleus of the forelimb muscles from the corticospinal tract on the side caudal to lower spinal cord injury in monkeys [15]. Furthermore, one study reported that the course of the corticospinal tract largely changed from the unaffected side to the affected side in such CNS regions as the cerebrum, brain stem, and cervical spinal cord following unilateral damage to the corticospinal tract [6]. Thus, it seems likely that, in animals paralyzed due to cervical spinal cord injury, restoration of motor function occurs because of both increased fibers from the spinal cord on the unaffected side and a change in the course of the corticospinal tract on the unaffected side.

In our study, a detailed examination of the restoration of the motor function in terms of the ROM test data showed a noticeable recovery of cubital joint motion (juveniles, 88.9 ± 20.6%; adults, 70.0 ± 29.2%) compared to carpal joint motion (44.4 ± 24.1% and 40.0 ± 21.1%, resp.). Furthermore, marked recovery in the motion of the cubital joint was observed in both juvenile and adult rats, while that of the carpal joint was less noticeable in both age groups; thus, there was a significant difference between cubital joint motion recovery and carpal joint motion recovery (*P* < 0.05). Besides the aforementioned possible involvement of the corticospinal tract on the unaffected side, a primate experimental study demonstrated the existence of a nerve pathway called the C3-C4 propriospinal neuronal pathway on the ventral aspect of the lateral funiculus that projects onto the C6-C7 motorial nucleus [16]. The existence of the C3-C4 propriospinal neurons has also been demonstrated in rats [17], and one previously published report stresses the profound importance of the C3-C4 propriospinal neurons, especially in recovery of manual motor function [18]. Taken together, these findings suggest that, in the present study, the less pronounced restoration of motor function in the carpal joint relative to the cubital joint was attributed to the resection of the C3-C4 propriospinal neurons along with the cervical spinal cord hemisection. Thus, the results of our study emphasize the strong bearing of the compensatory mechanism of the unaffected side on the restoration of cubital joint motion, whereas the presence of the C3-C4 propriospinal neurons was necessary for the restoration of carpal joint function.

In addition, the comparison of motor function recovery between juvenile and adult rats revealed that recovery of the FLS score was more pronounced in the juvenile rats and that recovery of cubital joint motor function, as assessed by the New Rating Scale and in terms of the ROM, differed significantly between adult and juvenile rats. This is corroborated by the long-standing concept that, according to the Kennard principle based on studies on brain nerve damage in monkeys, neural compensatory mechanisms operate more effectively in juvenile brains than in adult brains [2]. In addition, a study using cats demonstrated that restoration of motor function after spinal cord injury was more pronounced in juvenile cats than in adult cats, presumably via restriction of nerves functioning in an unnecessary manner in the juvenile animal group [19]. Therefore, it is possible to assume that juvenile rats in this study experienced the formation of a new nerve tract network and neurofunctional restriction. Furthermore, it has been demonstrated that the younger the rat, the greater the increase in serotonin and other neurotransmitters after spinal cord injury; this trend has a significant impact on the more pronounced restoration of motor function in juvenile rats as compared to adult rats [20]. Thus, it is highly conceivable that motor function compensation from the unaffected side is in fact greater, likely due to the formation of a new nerve tract network and/or an increased release of neurotransmitters in juvenile rats compared to in adults. If this were the case, then this would explain the greater degree of restoration in motor function observed in this study. Although motor function was estimated to have recovered because of the remaining corticospinal tract and activation of neurotransmitters, anatomical and electrical verification of the compensatory altered nerve networks and sprouting nerves are needed.

## 5. Conclusion

In the current study, the motor function of both forelimbs after precise spinal cord hemisection was evaluated in juvenile and adult rats over a given time. Immediately following the hemisection, rats presented with motor paralysis on the affected side alone. We observed that motor function restoration in the affected forelimbs tended to be more efficient in juveniles than in adult rats. Moreover, we determined that motor function compensation from the unaffected side played a crucial role in juveniles, but not in adult rats, resulting in a greater degree of restoration in motor function. In future research, an alteration of the remaining spinal cord axons, especially corticospinal neurons and reticulospinal neurons, should be closely examined using neuroanatomical and neurophysiological study. We are convinced that these results will help lead to a positive implementation in regenerative medicine.

## Figures and Tables

**Table 1:** Forelimb Locomotor Scale (FLS): methods of scoring.

Score	Contents
0	No movements of the forelimb (shoulder, elbow, or wrist joints)
1	Slight movements of one or two joints of the forelimb
2	Extensive movement of one joint and slight movement of another joint of the forelimb
3	Slight movement of all three joints of the forelimb
4	Extensive movement of one joint and slight movement of two joints of the forelimb
5	Extensive movement of two joints and slight movement of one joint of the forelimb
6	Extensive movement of all three joints of the forelimb
7	Plantar placement of the forelimb with no weight support
8	Dorsal stepping only
9	Dorsal stepping and/or occasional plantar stepping
10	Frequent plantar stepping
11	Continuous plantar stepping
12	Continuous plantar stepping with paw position rotated (at either initial contact, lift-off, or both)
13	Continuous plantar stepping with paw position parallel (at either initial contact, lift-off, or both)
14	Continuous plantar stepping with paw position rotated (at either initial contact, lift-off, or both) and occasional toe clearance
15	Continuous plantar stepping with paw position parallel (at either initial contact, lift-off, or both) and occasional toe clearance
16	Continuous plantar stepping with paw position parallel (at either initial contact, lift-off, or both) and frequent toe clearance
17	Continuous plantar stepping with paw position parallel (at either initial contact, lift-off, or both) and continuous toe clearance
